# Baby’s Rights

**Published:** 1896-06

**Authors:** 


### Baby's Rights.

Babies are human, and, like grown-up human beings, have their rights. But few people appear to think so. How would any man enjoy having nearly every one who entered his house tickle him in the ribs and "keechee" at him and not expect him to protest against such treatment. Yet in such way are most babies treated to make them laugh ; and because they are unable to speak they have to endure it. Babies should be kept free from all nervous excitement and as quiet as possible. They should not be made to laugh immoderately, because it induces wind to gather within the stomach, and many a violent fit of crying is occasioned thereby.

When our second child was an infant of some five months, we had, one day, a house full of company. We were busied with waiting upon them, and the baby was passed about from one to another, each guest trying to outdo the last in making him laugh.

We did not find time to pay much attention to our child until the company had left, when we found him limp and weak, pulse a feeble flutter, and a cold perspiration covering his body.

The babe had not been fed for some time, but would not nurse, only lying perfectly motionless, with wide-open eyes. Becoming alarmed, a doctor was called, who said, "The child has the appearance of having passed through some undue excitement, which has brought on nervous collapse."

We told the physician the events of the afternoon, and he thought that sufficiently accounted for the baby's condition. After that night of anxious watching and our baby's slow recovery, we forbade all tickling and undue tossing of him.

Perhaps few children would have been thrown into the same condition, yet those who are of the nervous temperament should be protected against such danger.

